# Assessing Performance of Bayesian State-Space Models Fit to Argos Satellite Telemetry Locations Processed with Kalman Filtering

**DOI:** 10.1371/journal.pone.0092277

**Published:** 2014-03-20

**Authors:** Mónica A. Silva, Ian Jonsen, Deborah J. F. Russell, Rui Prieto, Dave Thompson, Mark F. Baumgartner

**Affiliations:** 1 Center of the Institute of Marine Research (IMAR) and Department of Oceanography and Fisheries, University of the Azores, Horta, Portugal; 2 Laboratory of Robotics and Systems in Engineering and Science (LARSyS), Lisbon, Portugal; 3 Biology Department, Woods Hole Oceanographic Institution, Woods Hole, Massachusetts, United States of America; 4 Department of Biology, Dalhousie University, Halifax, Nova Scotia, Canada; 5 Sea Mammal Research Unit, Scottish Oceans Institute, University of St. Andrews, St. Andrews, United Kingdom; 6 Centre for Research into Ecological and Environmental Modelling, University of St. Andrews, St. Andrews, United Kingdom; University of Manchester, United Kingdom

## Abstract

Argos recently implemented a new algorithm to calculate locations of satellite-tracked animals that uses a Kalman filter (KF). The KF algorithm is reported to increase the number and accuracy of estimated positions over the traditional Least Squares (LS) algorithm, with potential advantages to the application of state-space methods to model animal movement data. We tested the performance of two Bayesian state-space models (SSMs) fitted to satellite tracking data processed with KF algorithm. Tracks from 7 harbour seals (*Phoca vitulina*) tagged with ARGOS satellite transmitters equipped with Fastloc GPS loggers were used to calculate the error of locations estimated from SSMs fitted to KF and LS data, by comparing those to “true” GPS locations. Data on 6 fin whales (*Balaenoptera physalus*) were used to investigate consistency in movement parameters, location and behavioural states estimated by switching state-space models (SSSM) fitted to data derived from KF and LS methods. The model fit to KF locations improved the accuracy of seal trips by 27% over the LS model. 82% of locations predicted from the KF model and 73% of locations from the LS model were <5 km from the corresponding interpolated GPS position. Uncertainty in KF model estimates (5.6±5.6 km) was nearly half that of LS estimates (11.6±8.4 km). Accuracy of KF and LS modelled locations was sensitive to precision but not to observation frequency or temporal resolution of raw Argos data. On average, 88% of whale locations estimated by KF models fell within the 95% probability ellipse of paired locations from LS models. Precision of KF locations for whales was generally higher. Whales’ behavioural mode inferred by KF models matched the classification from LS models in 94% of the cases. State-space models fit to KF data can improve spatial accuracy of location estimates over LS models and produce equally reliable behavioural estimates.

## Introduction

The collection of individual animal movement data has become widely utilized by ecologists in the last decade due to the improvement of the underlying technologies and reduction of operational costs involved in animal telemetry. Of the several technologies available, one of the most popular is that based on satellite tags (platform transmitter terminals, PTTs) using the Argos system [1]. However, most satellite tags record observations at irregular intervals and with considerable error, meaning that movements are observed neither continuously nor with complete accuracy. The Argos service provider assigns a quality index, or location class (LC), to each position based on its estimated precision. The radius of error (assumed to include 68% of positions) for each LC is: LC 3<250 m, LC 2 250–500 m, LC 1 500–1500 m, LC 0>1500 m, and LC A, B and Z for which no estimate of error is provided [2]. However, attempts to measure spatial error of Argos locations using either stationary tests or double-tagging experiments with free-ranging animals consistently reported larger errors than those indicated by Argos. Many of these studies also provided error estimates for location classes A and B, showing these could be in the range of tens to hundreds of kilometres (reviewed in [3]).

Varying accuracy and precision, and unevenness in space and time of telemetry data can affect the determination of distribution, habitat use and behavioural patterns of animals and severely bias the calculation of movement metrics [3]–[5]. Therefore, advanced statistical methods are necessary to account for spatial error and temporal irregularity in the data and to understand the movement behaviour of the tracked animals.

Jonsen et al. [6] proposed a state-space framework for analysis of movement data that was further developed in Jonsen et al. [7]–[9], in order to deal with the biological and statistical complexities associated with animal tracking data. State-space models (SSMs) offer a powerful way to infer latent movement from imperfect estimates of animal locations by allowing uncertainty in both the observations and in the movement dynamics to be accounted for separately in the estimation process. Additionally, movement models can include behavioural or environmental effects, enabling a better understanding of the interaction between an animal’s behaviour and its environment [8]–[11]. SSMs have been used widely among ecologists and are currently one of the tools of choice for analysing tracking data of several taxa and across environments [5], [12]–[17].

Geolocation of animals tracked with Argos systems is based on the Doppler shift of the tag’s fixed transmission frequency; i.e. the frequency shift of the tag’s signal received at the orbiting satellite as it approaches and moves away from the tag [18]. The system estimates two possible positions, which are symmetrical on each side of the satellite ground track. Until recently, Argos used a non-linear Least Squares (LS) algorithm to refine the tag’s position estimates and to select the one with the minimal residual error. However, the LS positioning algorithm presented a number of limitations and affected the quality of the tracks obtained. For instance, when the LS algorithm could not complete the refinement routine or check the validity of the most plausible location estimate, no position was provided. In addition, the process required at least two transmissions (also called messages) during a single satellite pass to compute a position and at least four messages to produce an error estimate.

In May 2011, Argos implemented a new algorithm that accounts for movement dynamics and uses a Kalman filter (KF) to estimate positions [1], [19]. The algorithm uses a correlated random walk model to predict the next position and its estimated error based on the previous positions and estimated error. It then uses the Doppler frequency-shift measurements acquired during a satellite pass to update the position predicted by the model and return a final position. Compared to the LS method, the Kalman filtering estimator is reported to improve the accuracy of estimated positions and to increase the number of positions up to 13% [19]. Such improvements may have a significant impact in studies where relatively few messages are received with each satellite pass, which is the case for many marine and dense forest species.

Although the new processing algorithm may bring significant advantages, it may also introduce changes in the autocorrelation structure of the Argos satellite data. Given that many published SSM applications for animal tracking data do not currently account for the potential autocorrelation in location errors introduced by the new KF algorithm, models fit to datasets with differing degrees of autocorrelated errors could lead to biased estimates of movement parameters, behavioural states, and their uncertainties. Several studies have examined the validity of SSMs applied to data obtained with the LS positioning algorithm and quantified the precision of predicted locations (e.g. [20]–[22]), but to the best of our knowledge, no study examined how changes introduced by the KF algorithm might affect the application of these models.

Our aim is to assess the performance of Bayesian SSMs fit to satellite tracking data processed with the new KF positioning algorithm introduced by Argos. We use two real datasets from marine taxa that differ greatly in their movement ranges– harbour seals (*Phoca vitulina*) and fin whales (*Balaenoptera physalus*) – as SSMs are known to be sensitive to the scale of movement [20]. Using data from 7 harbour seals instrumented with ARGOS satellite transmitters equipped with Fastloc GPS loggers (hereafter GPS/Argos tags), we compared estimated locations from a hierarchical SSM (hSSM) fit to data processed with KF and LS algorithms to the GPS positions obtained from the same tag to (1) assess spatial accuracy of locations from models fit to data derived from each algorithm; and (2) determine how spatial accuracy varies with observation frequency, temporal resolution and reported precision of Argos locations. Models fit to fin whale tracks could not be evaluated through comparison with GPS data because whales were instrumented with Argos-only transmitters. Satellite tracks of 6 fin whales were used to compare location and behavioural states estimated from a switching state-space model (SSSM) fit to the KF data to those from models fit to the classical LS algorithm. We analysed whale tracks with different temporal resolutions to test whether and how the quality of tracking data affected the similarity of the output from SSSMs fit to LS and KF data.

## Materials and Methods

### Ethics Statement

All seal handling and tagging procedures were carried out under license number 60/4009 issued by the UK Home Office under the Animals (Scientific Procedures) Act 1986.Whale tagging was approved by the Regional Directorate for Sea Affairs, Autonomous Region of the Azores under research permits 20/2009/DRA and 16/2010/DRA. All procedures in whales followed the guidelines of the American Society of Mammalogists [23].

### Data Collection and Processing

In the interest of clarity we’ll use the following terminology throughout the paper: i) LS locations/data and KF locations/data refer to the locations/data provided by Argos that were derived from the application of the LS and KF algorithms, respectively; and ii) LS or KF model refer to the state-space models fit to data derived from the application of either the LS or KF algorithm. As explained below, the same models were fit to LS and KF datasets.

#### Harbour seal data

GPS/Argos tags were deployed on harbour seals in the Eden Estuary, south-east Scotland and around Eday, Orkney between May and July 2012. Animals were caught on or close to haul-out sites using hand, seine or tangle nets and subsequently anesthetised with Zoletil as detailed in Sharples et al. [24]. Tags were attached to the fur at the back of the neck using Loctite 422 Instant Adhesive. Tag duration ranged from 25 to 65 days (median 41 days).

The Fastloc GPS data used in this study were transmitted via the Argos system, providing high resolution at sea locations. The Argos transmissions also generated a concurrent series of standard Argos locations. At our request, messages from the satellite transmitters were processed by the Argos service provider (CLS, Ramonville Saint-Agne, France) using both the LS and KF processing algorithms.

Fastloc GPS positions are more accurate and precise than Argos locations and in the present study were assumed to represent the seals’ “true” position. However, GPS accuracy is known to decrease when Fastloc calculations are based on fewer satellites [25], [26] and when residual error is high [27]. GPS data were therefore cleaned according to the Sea Mammal Research Unit protocol where locations estimated with <5 satellites and with residual errors = 0 or >25 were removed [27]. Tests on land showed that over 95% of the cleaned locations had an error of <50 m [27].

As central-place foragers harbour seals haul-out on land between foraging trips. Thus, we needed to remove haul-out locations from the data before fitting any models. Although the GPS/Argos tags have a wet/dry sensor which records haul-out events, only a subset of these records are received via the Argos system. These animals often range in near shore waters and the large measurement error in Argos observations means such observations could not be used to define whether a location fell on land. Thus, we used the Fastloc GPS positions to define the precise time seals departed and returned to land. Positions within 200 m from all shorelines were also considered as haul-out to buffer against errors in GPS positions and because harbour seals haul-out on intertidal sandbanks. This procedure may have excluded valid parts of a few foraging trips but this shouldn’t affect algorithm comparison in anyway. Consecutive at sea locations between haul-out events thus formed an individual foraging trip. We defined a series of trips within each seal GPS track and, for each trip, we selected all LS and KF locations obtained between 5 minutes prior to and 5 minutes after the trip. Only trips with ≥30 LS and KF locations were subsequently used for model fitting. The seal dataset analysed in the next sections consisted of 1174 GPS, 1339 Argos LS and 2083 Argos KF positions obtained during 31 foraging trips of 7 different seals (Table S1).

#### Fin whale data

The data consisted of Argos-derived surface positions obtained from PTTs (model SPOT5-implantable, Wildlife Computers, Redmond, Washington, USA) attached to the flanks of 6 fin whales. Whales were tagged off Faial and Pico islands (38°N 28°W), Archipelago of the Azores (Portugal), in September 2009, April and May 2010. All tags were programmed to transmit on a daily basis, every hour of the day up to a maximum of 500 messages per day. Details about the tagging methodology, movements and inferred behaviours of these whales are described in Silva et al. [28]; here we focus on the analyses of model fitting and performance. Like in the case of the harbour seal data, we requested location data to be processed with both the LS and KF algorithms.

The KF algorithm consistently yielded more positions per individual whale than the LS algorithm (Table S2). To compare the regular, estimated locations from the LS model with those from the KF model for each whale dataset, we selected only the positions from the KF data that were within 2 minutes of a LS position (hereafter called the KF reduced dataset).We fitted a second model to all KF locations to investigate how the tracks from a model fitted to the full KF dataset compared to those from a LS model.

### State-space Models

State-space models couple two stochastic models: a process model (transition equation) that estimates the current state (e.g. location and behavioural state) of an animal given its previous state, and an observation model that relates the unobserved location states estimated by the process model to the observed data (locations obtained from Argos).

The SSM described in Jonsen et al. [8] uses a first-difference correlated random walk (DCRW) as the process model to describe movement dynamics. The SSSM also uses a DCRW as the process model but allows movement parameters to change between two discrete behavioural states – for example, transiting versus area-restricted search (ARS; [29]) – by including a different DCRW model for each [9].

#### Model fit to harbour seal data

We initially attempted to fit a SSSM to the harbour seal data but encountered the same problems noted by Breed et al. [20] using simulated tracks. These authors showed that when the scale of movement is small relative to observation error and frequency, the models are unable to accurately estimate location and behavioural states. Even though the temporal resolution of our seal data was reasonably high (see Table S1), the SSSM provided a poor fit, resulting in unreliable location and behavioural estimates, irrespective of the algorithm used (although models fitted to KF data behaved slightly better). It is possible that movements of harbour seals are best analysed with different models (e.g. [30]) but this evaluation is beyond the scope of this paper.

We therefore chose to fit a SSM [8] to the harbour seals’ satellite locations derived from the LS and KF algorithms. The SSM was fit as a single hierarchical model (hSSM) [5] to all trips of all seals simultaneously, as this significantly improved parameter estimation, especially for data-sparse trips.

By letting *k* index each individual harbour seal trip, the transition equation of the SSM formulated within a hierarchical framework becomes:
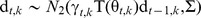
where **d** *_t_*_-1_ is the displacement between unobserved locations **x** *_t_*_-1_ and **x** *_t_*_-2,_ and **d** *_t_* is the displacement between unobserved locations **x** *_t_* and **x** *_t_*_-1_. **T**(θ) is a transition matrix that provides the rotation required to move from **d** *_t_*_-1_ to **d** *_t_*, where θ is the mean turning angle. γ is the move persistence coefficient (i.e. combined autocorrelation in direction and speed). *N*_2_ is a bivariate Gaussian distribution with covariance matrix ∑ and represents the randomness in animal movement.

The observation equation accounts for the irregularity and variable errors in the observed Argos locations. Errors in latitude and longitude are modelled with a t-distribution using independent parameter estimates derived for each Argos location class [8], [31]. We fitted the same observation equation to data processed with LS and KF algorithms. Further details about the SSM are provided in Jonsen et al. [5], [8].

#### Model fit to fin whale data

We fitted the Bayesian switching state-space model (SSSM) described in Jonsen et al. [9] to the Argos satellite-based location estimates of fin whales derived from the LS and KF algorithms. The transition equation for the SSSM is similar to that of a SSM:
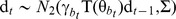
but in this case the movement parameters θ and γ are indexed by behavioural state *b*. At each displacement *t*, the estimated behavioural state *b* corresponds to the set of parameters θ and γ that provide the best model fit.

The observation equation used to model the irregularly observed LS and KF fin whale locations was that same used for the SSM.

### Model Implementation

Models were fit using R (R Development Core Team 2012) code provided in the supplement to Jonsen et al. [5]. The code implements hSSM and SSSM using Markov Chain Monte Carlo (MCMC) methods using the program Just Another Gibbs Sampler (JAGS).

The hSSM was fitted separately to the harbour seals’ location data (excluding Z class locations) obtained from each algorithm using a time step of 2 hours, corresponding to the average temporal resolution of the LS data. For the hSSM fit to the KF and LS satellite datasets, we ran two MCMC chains for 60000 iterations, dropping the first 50000 samples as a burn-in and retaining every 10^th^ sample from the remaining 10000 assumed post-converge samples from each chain to reduced sample autocorrelation. Thus, model parameters and estimates of seals’ locations were calculated using a total of 2000 MCMC samples.

The SSSM was fitted separately to the fin whales’ data obtained from each algorithm (after removing Z class positions from both datasets) using a time step of 3 hours, corresponding to the average temporal resolution of the LS data. For each SSSM we ran two MCMC chains for 45000 iterations, discarding the first 40000 samples and retaining every 5^th^ from the remaining 5000 samples from each chain. A total of 2000 MCMC samples were used to calculate model parameters and estimates of whales’ locations and behaviours.

hSSM and SSSM convergence and sample autocorrelation were assessed by visually inspecting trace and autocorrelation plots and using the Gelman and Rubin scale reduction factor (R-hat) diagnostic available in R package boa.

### Data Analysis

The Argos locations per seal trip greatly exceeded those of Fastloc GPS, and the latter were also more irregular in time (Table S1). Therefore, in order to estimate the accuracy of locations predicted by LS and KF models, we first selected only those locations that were within 30 min of a GPS position. We then estimated the “true” position of the seal at the time of those modelled locations by linear interpolation between two consecutive GPS positions [32]. Finally, we calculated the linear error and absolute latitudinal and longitudinal errors between each modelled location and the corresponding interpolated GPS position.

To investigate if and how the quality of Argos telemetry data affects spatial accuracy of LS and KF models, we compared location errors from seal trips with different temporal resolutions, spatial precisions and frequency of observations. We used linear mixed-effects models with seal and individual trip as random effects to account for behavioural differences among seals and unequal sample sizes across trips. Errors were log transformed to ensure linearity with continuous predictors. Algorithm (LS vs. KF) was included in the model as a categorical predictor and continuous predictors were number of Argos locations used to fit the model, average length of time between locations (hereafter time step), and proportion of positions of LC 0, A and B (hereafter LC 0-B). Values of these continuous predictors for each seal trip are given in Table S1. We fitted a model with interactions between algorithm and all continuous predictors because we were interested in investigating if the effect of data quality was consistent among the LS and KF models.

In the case of the SSSM fit to the whale data, we could only determine how well the KF models performed in relation to models fit to the LS algorithm. For each whale, we compared the medians, inter-quartiles and 95% credible limits (95% CL) of parameter estimates of LS and KF models. We also calculated the longitudinal and latitudinal differences between pairs of location estimates from the LS and KF models for each whale. For each location predicted by the LS model we estimated a probability ellipse determined by the 95% CL obtained from the model. We then calculated the proportion of location estimates from the reduced KF model that fell within the 95% probability ellipse of the corresponding LS position.

To understand if the KF algorithm introduced significant changes in the ability of the SSSM to resolve behavioural state, we calculated percentage of agreement in behavioural classification between the LS and KF models. Whale behaviour at each 3-h location was inferred from the output of the SSSM. Because behaviour is treated as a binary variable, MCMC samples can only assume the values 1 (inferred as transiting) or 2 (inferred as ARS), *b* at each location was estimated as the mean value of the MCMC samples. We used the same cut off points as Jonsen et al. [9]: locations with mean estimates of *b*<1.25 were assumed to represent transiting, *b*>1.75 ARS, and between these values were considered “uncertain”.

Finally, we investigated how the whale tracks from a model fitted to the full KF dataset compared to those from the models applied to LS data. We fitted the SSSM to the full KF data using the same time step as above. For each whale we calculated the distance (in km) from locations estimated by the full KF model to the track estimated by the LS model. We compared only data from days when both methods delivered satellite locations.

Means are presented ± standard deviation (SD) throughout. All distances were calculated using a great-circle route. Statistical analyses were performed in R software using packages nlme and MASS.

## Results

### Accuracy and Precision of LS and KF Models Fit to Harbour Seal Data

The KF algorithm provided 2083 locations, 1.5 times more than the LS algorithm and 1.8 times more than the GPS transmitted via Argos (Table S1). The increase in the number of locations per trip in relation to the LS data ranged from 12 to 137% with an average of 56%. A total of 368 LS and 375 KF model locations were within 30 min of a GPS position and were used to compare spatial accuracy of locations derived from each hSSM (Table 1).

**Table 1:** Errors in locations estimated from models fit to Least Squares (LS) and Kalman filtered (KF) data for all harbour seal trips.

Seal	Trip	LS model			KF model			Variationin mean errors (%)*
		N†	Mean error (km)	Error range (km)	N†	Mean error (km)	Error range (km)	
1545	11	8	2.6±1.7	0.4–5.1	8	1.7±1.4	0.1–3.9	−34
	21	9	1.9±2.0	0.1–5.6	8	0.8±0.4	0.4–1.5	−57
	23	5	2.2±1.3	1.2–4.3	6	0.9±0.3	0.6–1.4	−56
	27	14	1.9±2.3	0.1–8.6	15	2.2±2.4	0.1–7.9	16
	28	13	1.5±2.1	0.1–7.6	13	1.5±1.5	0.1–5.1	−2
28503	11	12	2.2±1.9	0.2–6.9	14	2.0±2.3	0.2–8.5	−10
	18	12	3.9±2.4	1.7–9.4	11	3.1±2.7	0.3–9.3	−23
	19	14	4.5±3.1	0.2–10.5	14	4.4±3.4	0.8–13.9	−3
	23	8	4.8±1.8	1.6–6.8	8	4.2±2.9	0.9–8.9	−13
	42	5	2.9±1.7	1.0–5.5	6	2.4±1.1	1.1–3.8	−19
43844	4	15	2.5±2.6	0.1–10.0	14	1.6±1.6	0.1–5.7	−36
	8	8	1.7±1.5	0.2–4.6	8	1.2±0.6	0.2–1.9	−29
	14	10	1.7±2.4	0.1–8.0	12	1.4±1.7	0.1–6.4	−23
	16	13	1.5±1.5	0.2–6.3	11	1.0±0.7	0.2–2.3	−32
	22	14	4.9±3.7	0.9–13.6	14	2.5±2.7	0.2–9.0	−48
43871	7	24	3.6±3.5	0.3–12.7	23	3.4±3.4	0.3–11.7	−5
	8	17	5.9±3.6	0.8–11.8	17	4.3±3.2	0.7–10.1	−27
	13	21	4.9±3.7	0.3–10.8	20	3.0±2.5	0.1–9.4	−39
	19	20	5.9±2.9	0.6–11.5	20	4.5±2.8	0.2–10.6	−24
120346	24	13	1.9±1.4	0.5–5.6	13	1.2±0.7	0.3–2.6	−37
	25	15	2.2±1.7	0.4–5.4	15	1.6±1.3	0.2–4.1	−31
	26	5	4.3±2.9	1.3–8.4	5	3.0±3.2	0.9–8.7	−30
	30	13	2.1±1.4	0.4–4.9	13	1.1±1.0	0.1–3.2	−48
	32	7	1.7±1.4	0.6–4.1	7	1.7±1.9	0.2–5.9	−1
120349	3	8	4.2±2.3	1.2–7.1	8	4.5±3.5	0.6–10.8	6
	4	8	3.5±2.8	0.4–7.9	8	4.2±3.9	0.9–12.2	20
	5	5	3.7±1.7	2.0–6.1	5	1.9±1.7	0.8–5.0	−48
	6	10	7.7±3.5	1.1–11.1	10	5.0±3.0	0.5–8.8	−35
120350	3	13	3.7±3.1	0.5–9.4	13	3.1±2.3	0.1–3.9	−16
	4	14	3.2±3.1	0.3–12.5	13	5.0±4.5	0.4–1.5	56
	5	15	4.2±2.4	0.7–7.5	23	4.9±4.3	0.6–1.4	17
Total		368	3.5±3.0		375	2.9±2.9		

*Variation in mean errors was calculated for each trip as the difference in the mean error estimated for KF and LS models divided by the mean error of the LS model.

† N: Number of locations used to calculate errors in locations estimated from LS and KF models.

**Figure 1:**
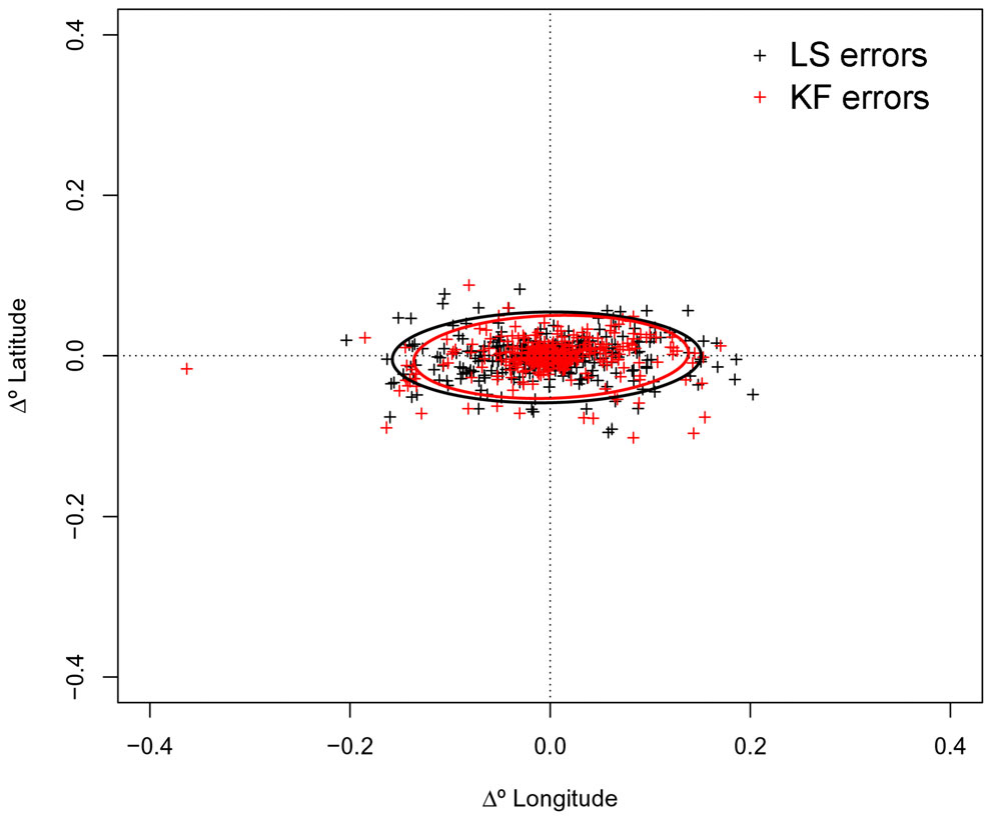
Errors in locations estimated from LS and KF models. Errors in harbour seal locations estimated from state-space models fit to Least Squares (LS) (black) and Kalman filtered (KF) (red) data are plotted as offsets from “true” GPS positions. Standard ellipses were fitted to 95% of LS (black line) and KF (red line) error points.

Errors in locations estimated from LS and KF models showed the same elliptical distribution in relation to interpolated GPS positions, with a clear directional bias in the longitudinal error component (Fig. 1). Average longitudinal errors ranged between −0.20°–0.20° (mean = −0.003) for LS models and between −0.36°–0.17° (mean = 0.001) for KF models. Latitudinal errors ranged between −0.10°–0.08° (mean = −0.002) for LS models and between −0.10°– 0.09° (mean = 0.001) for KF models. Overall, the mean distance of KF model locations to interpolated GPS positions was lower (2.9±2.9 km) than that of LS model locations (3.5±3.0 km) (Table 1). About 31% of all locations predicted from the KF model were within 1 km from the interpolated GPS position and 82% were less than 5 km. For locations predicted from the LS model, 24% and 73% were respectively within 1 km and 5 km from the corresponding interpolated GPS position. The KF model produced smaller mean errors for 27out of 31 trips (Table 1). Predicted trips from the KF model were 27% (range: 1–57%) more accurate than trips derived from the LS model. However, standard deviations of KF errors were sometimes higher suggesting that location accuracy varied considerably within the same trip (Table 1). Average errors of trips increased as the average distance between locations (step length) increased. For trips with an average step length ≥6 km, the average error of KF modelled locations was 4.6±0.4 km, and of LS modelled locations was 5.9±1.2 km.

**Figure 2:**
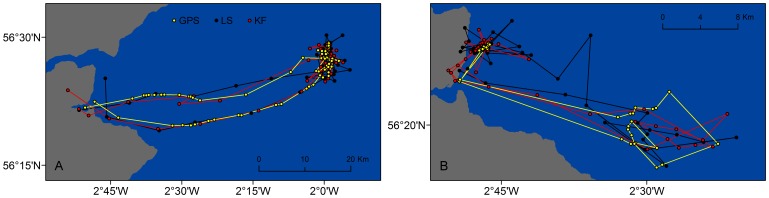
Harbour seal tracks obtained from GPS (yellow), LS (black) and KF modelled (red) locations. Estimated locations (circles) and tracks (lines) of harbour seals obtained from fitting state-space models to Least Squares (LS) (black) and Kalman filtered (KF) (red) data, in relation to the “true” GPS positions and track (yellow). A. Example of a trip with higher quality of Argos data: trip 7 of harbour seal #43871. B. Example of a trip with lower quality of Argos data: trip 22 of harbour seal #43844.

Two representative tracks of foraging trips reconstructed using GPS positions, and LS and KF modelled locations are shown in Fig. 2. In general, modelled tracks closely matched the GPS tracks, especially during periods of directed movement. Yet, tracks predicted by the LS model occasionally diverged greatly from the GPS track and tended to extend over a wider area in periods of torturous movements.

Uncertainty in KF model estimates, as indicated by the width of the 95% CL (measured in km), was significantly lower than that of LS model estimates (KF model: 5.6±5.6 km; LS model: 11.6±8.4 km; t-test = −11.41, df = 741, *P*<0.001).

### Effect of Data Quality on Accuracy of LS and KF Models Fit to Harbour Seal Data

**Figure 3:**
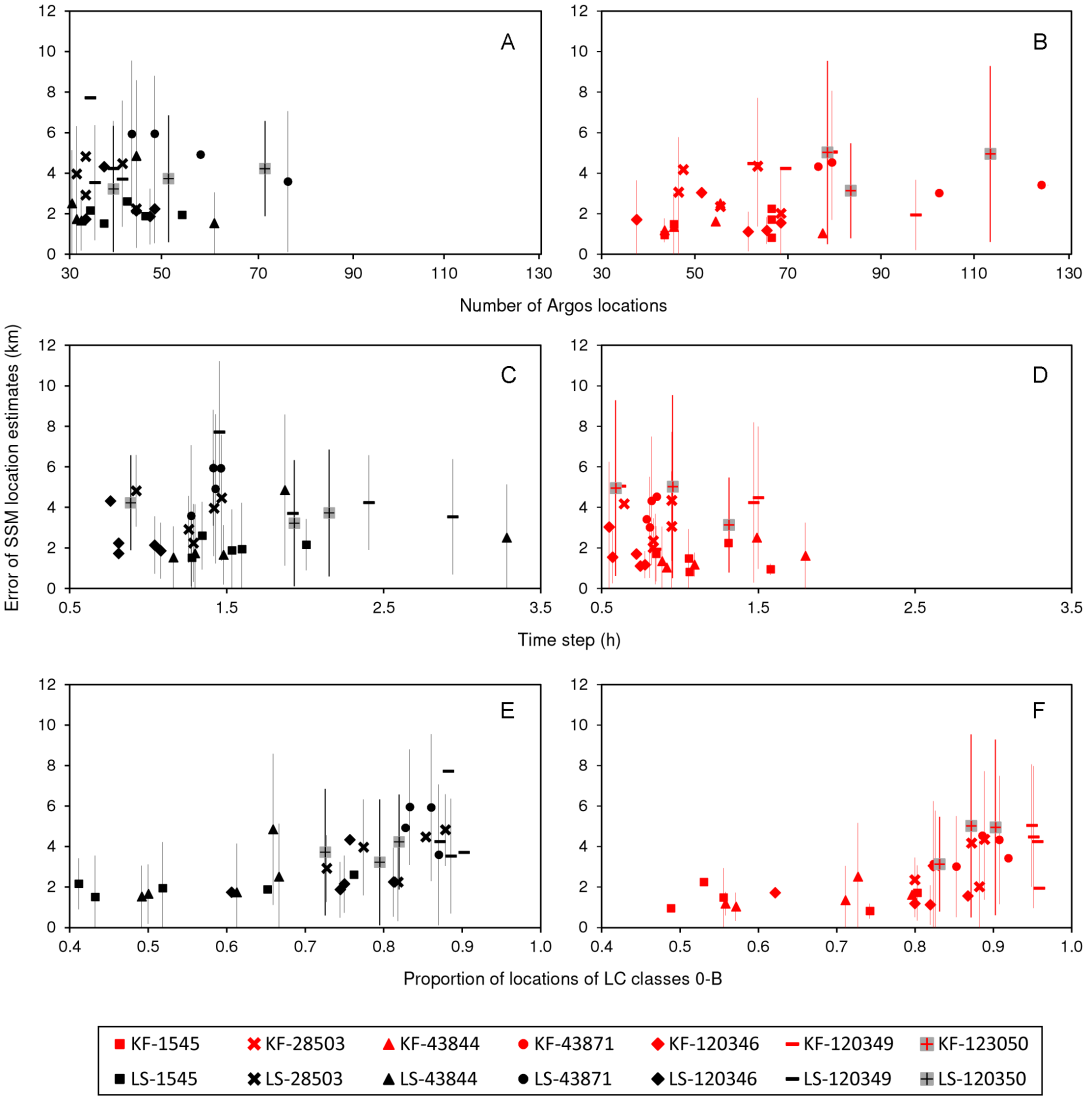
Trip-averaged error in locations estimated from LS and KF models relative to Argos data quality. Relationship between mean errors (±SD shown as vertical bars) in locations estimated from state-space models fit to Least Squares (LS) (black) and Kalman filtered (KF) (red) data per harbour seal trip and quality of Argos telemetry data used to fit the models: A–B. Number of locations. C–D. Time step (h) between locations. E–F. Proportion of locations of LC 0-B. Different trips from the same seal have the same symbol.

Observation frequency, temporal resolution and spatial precision of Argos data used to fit the SSMs varied among seals and trips and between the LS and KF models (Fig. 3, Table 1). Expectedly, the increase in number of locations that resulted from the application of the KF algorithm improved the temporal resolution of the KF data for all trips. However, it also increased the proportion of locations of lower spatial precision (Argos LC 0, A and B) in each trip. With few exceptions, trips from the same seal tended to have similar number of locations, time steps, and proportion of LC 0-B, suggesting an individual effect in the quality of Argos data. This could be due to tag (e.g. battery power), instrumentation (e.g. tag placement) or behavioural-specific (e.g. surface behaviour and diving time) differences among seals or to a combination of all these factors.

Mean errors (±SD) of LS and KF modelled trips were plotted in relation to the Argos quality parameters described above (Fig. 3). Accuracy of modelled trips did not seem to improve with the observation frequency or temporal resolution of Argos data, but mean errors (and respective SD) in LS and KF estimated locations appeared to increase with increasing proportions of locations LC 0-B.

**Figure 4:**
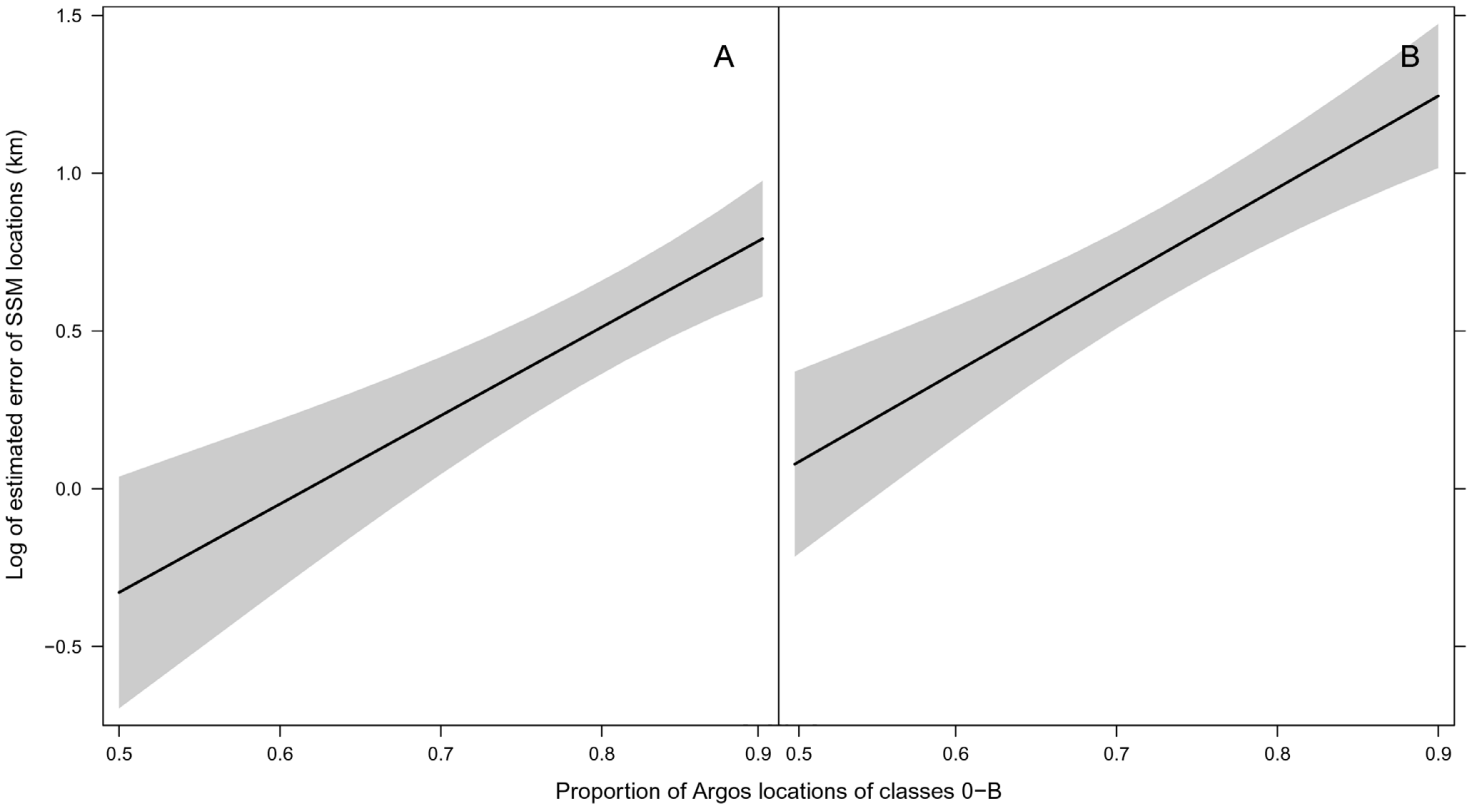
Predicted error in locations estimated from LS and KF models. Predicted error in harbour seal locations according to the best fitting linear mixed-effects model for A. State-space models fit to Kalman filtered (KF) data. B. State-space models fit to Least Squares (LS) data.

We fitted a linear mixed-effects model to examine the effects of type of algorithm and of Argos quality parameters (spatial precision, observation frequency and time step) on estimated errors of modelled locations. The interactions between algorithm and the continuous predictors were the first to be dropped from the linear mixed-effects model based on AIC results, suggesting that quality of Argos data influenced the accuracy of LS and KF models in a similar way. The best fitting model indicated that observation frequency and time step of Argos data had no effect on the errors of locations estimated from the models, and only algorithm and proportion of locations LC 0-B were significant (Table S3). Contrary to our expectations, there was little variability among different seals in addition to the trip-to-trip variability and both the AIC and the likelihood ratio test indicated that individual seal could be dropped from the model (*L* = 3.95×10^−7^, *P* = 0.499), leaving trip as the only random effect. The best fitting model predicted larger errors for locations estimated from LS models compared to locations from KF models (Fig. 4, Table S3). On average, LS models will estimate locations that are 1.6 km farther from the true seal position relative to KF locations. Also, errors (on a logarithmic scale) are expected to increase as proportion of Argos locations with lower precision increases, and this relationship was similar for LS and KF models (Fig. 4, Table S3).

### Comparison of LS and KF Models Fit to Fin Whale Data

Medians and 95% CL of estimated model parameters of the reduced dataset were similar across whales and between the LS and KF algorithms. Both the LS and KF models distinguished well between the two behavioural modes (transiting and ARS), as indicated by the parameter estimates that aggregated into two non-overlapping groups.

**Figure 5:**
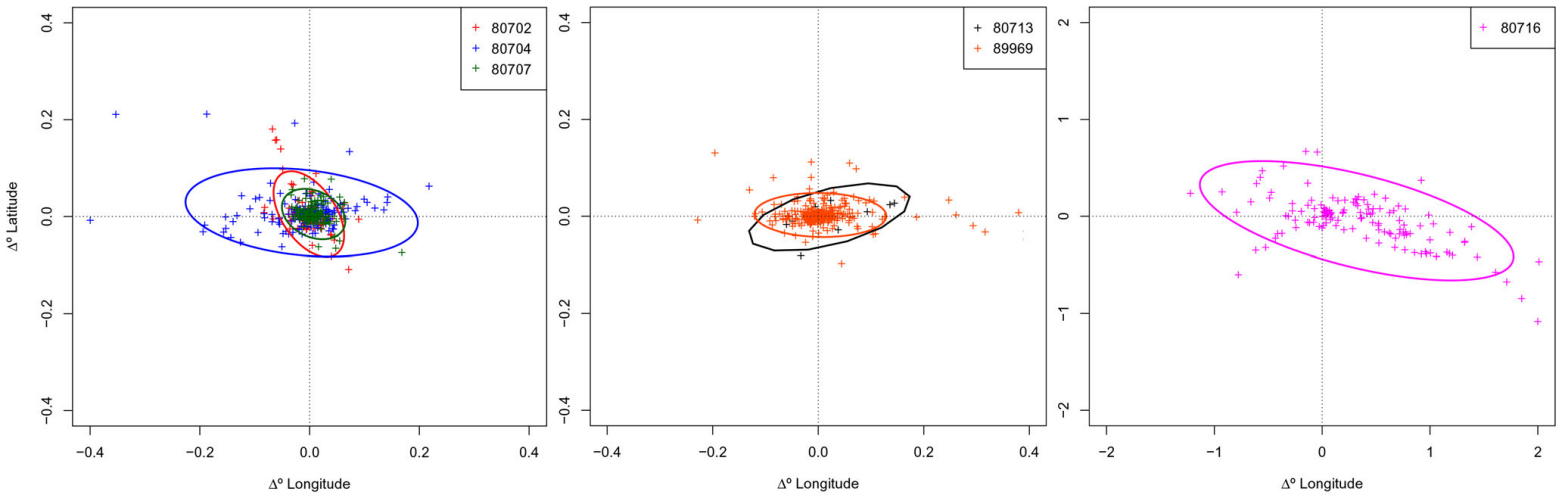
Differences in locations estimated from KF and LS models for all fin whales. Differences in locations estimated from switching state-space models fit to Kalman filtered (KF) (red dots) data are plotted as offsets from locations calculated from the same models fit to Least Squares (LS) data. Standard ellipses were fitted to 95% of KF data points. A. Fin whales #80702 (red), #80704 (blue) and #80707 (green). B. Fin whales #80713 (black), #89969 (orange). C. Fin whale #80716 (pink).

The estimated locations inferred from the KF model applied to the reduced dataset differed little from the locations output by the LS model. Differences in latitude and longitude between paired KF-LS locations were centred around zero but the latter showed a wider range of values (range for latitude: −1.1–0.7°; range for longitude: −1.2–2.0°) (Fig. 5). Differences in paired KF-LS locations were considerably higher for whale #80716. Removing data from this whale resulted in a considerable reduction in the range of latitudinal (−0.1–0.2°) and longitudinal (−0.5–0.4°) distances between KF and LS locations. Differences in latitude and longitude between paired locations showed no obvious trend with latitude, longitude, date, number of positions per track, or behavioural mode (not shown).

The proportion of estimated locations from the SSSM applied to the reduced KF data that fell within the 95% probability ellipse of locations inferred by the LS model varied between whales but was very high, ranging from 69 to 100% (mean = 88%). We also compared differences in the width (measured in km) of the 95% CL between pairs of locations estimated from the model fit to the reduced KF data and the LS data. For five whales, the reduced KF model resulted in lower average widths of 95% CL (paired t-test: *P*<0.05 for all whales), although differences were generally small (mean difference: −2.2±3.9 km). For whale #80716, however, the 95% CL of the reduced KF model were significantly wider than those of LS data (paired t-test: t = −11.15, *P*<0.001; mean difference: 76.2±80.3 km).

In 94% of the cases, the behavioural mode inferred by the KF model matched the classification from the model fit to the LS data (Table 2). Agreement was highest for locations inferred as transiting (98%), followed by ARS (93%). Changes in behavioural classification between the two models were from transiting or ARS to “uncertain” and vice-versa, but never from transiting to ARS or vice-versa.

**Table 2:** Agreement between fin whale behavioural modes inferred by models fit to Least Squares (LS) and Kalman filtered (KF) data.

		KF model		
		Transit	ARS*	uncertain
LS model	Transit	353	0	6
	ARS	0	524	40
	uncertain	5	6	83

The matrix shows the number of fin whale locations classified in each behavioural mode by the LS model that were assigned to each of the behavioural modes by the KF model.

*ARS Area restricted search.

As expected, the KF processing algorithm yielded more positions and improved the temporal resolution of the 6 whale tracks. The increase in number of locations per track ranged from 18 to 272% with an average of 75%. The average number of daily locations per whale track varied between 6.0–38.6 for the full KF data, compared to 1.6–30.8 for the LS data (Table S2). There was also an increase in track duration (3 and 11 days) for two whales but this came at the expense of a few gaps (maximum of 3 days) in those tracks (Table S2). In contrast, the KF algorithm provided several positions within a 5-day gap in the LS tracking data of whale #80716.

**Figure 6:**
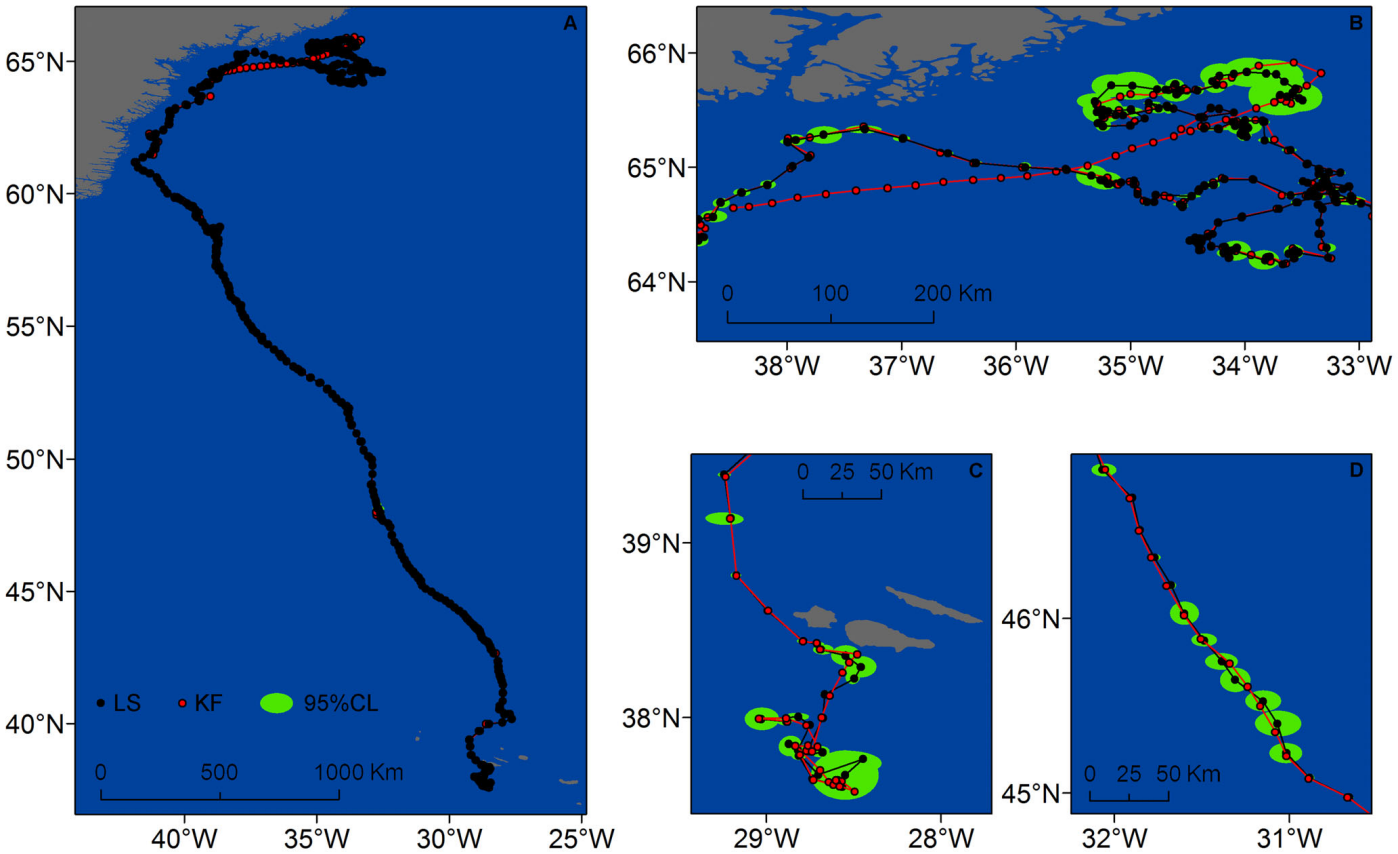
Fin whale #89969 tracks obtained from LS (black) and KF modelled (red) locations. Estimated locations (circles) and tracks (lines) of fin whale #89969 obtained from fitting a switching state-space model to Least Squares (LS) (black) and the full Kalman filtered (KF) (red) data. The 95% probability ellipses of locations derived from the LS-based model are shown in green. A. Complete tracks showing the increase in track length resulting from the application of the KF algorithm (red). B, C, D. Detail of the tracks showing the majority of KF locations within the 95% probability ellipses of LS locations.

**Figure 7:**
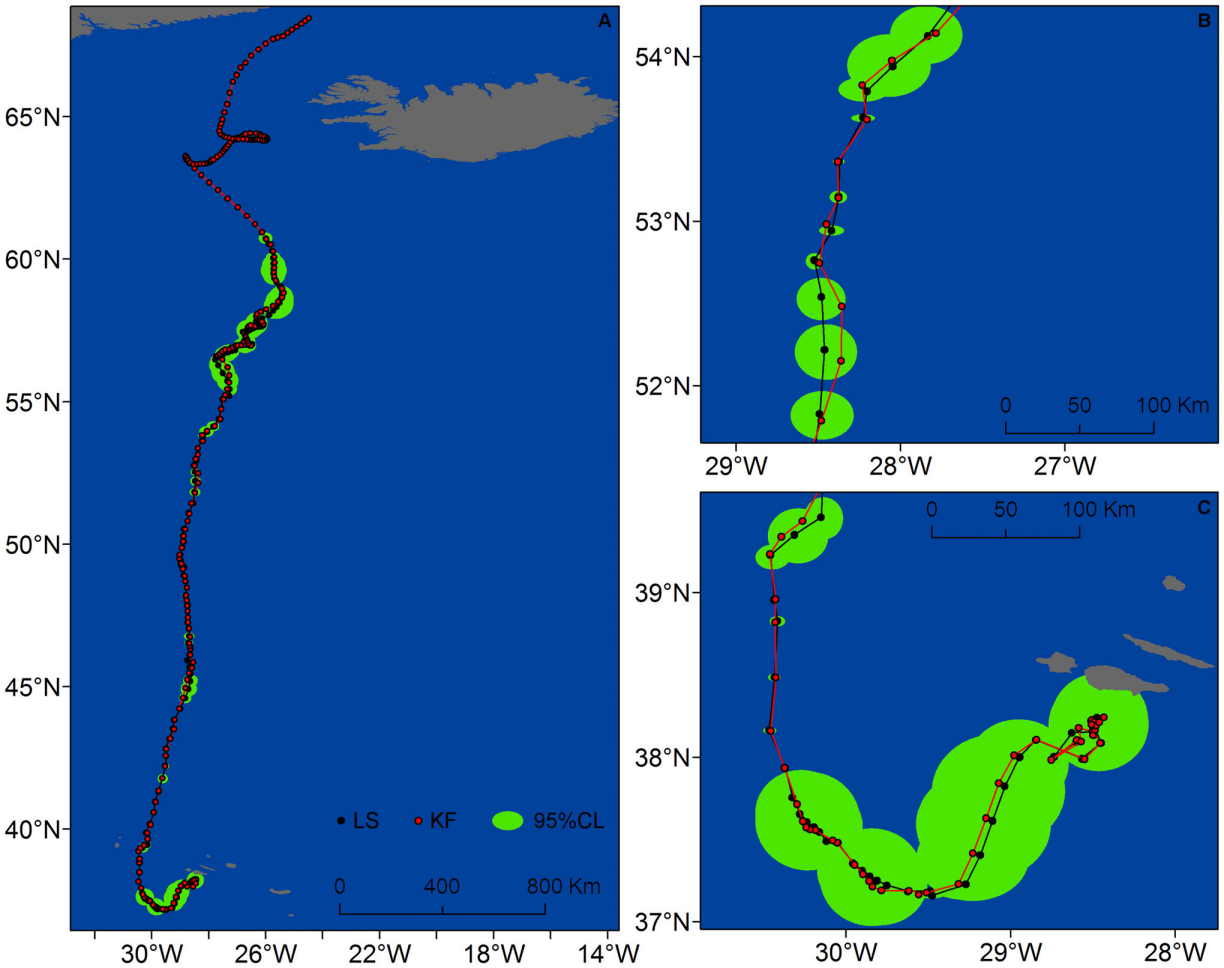
Fin whale #80704 tracks obtained from LS (black) and KF modelled (red) locations. Estimated locations (circles) and tracks (lines) of fin whale #89969 obtained from fitting a switching state-space model to Least Squares (LS) (black) and full Kalman filtered (KF) (red) data. The 95% probability ellipses of locations derived from the LS-based model are shown in green. A. Complete tracks showing the increase in track length resulting from the application of the KF algorithm (red). B, C. Detail of the tracks showing the majority of KF locations within the 95% probability ellipses of LS locations.

The width of the 95% CL of locations estimated by the full KF model (47.3±76.9 km) was significantly lower than the width of 95% CL of locations estimated from the LS model (57.2±113.0 km) (t = 2.38, *P* = 0.017). Still, locations from the full KF model fitted well the paths inferred from the LS data, except when gaps in the LS data exceeded 1 day (Figs. 6 and 7). Combining data from all whales, over 49% of locations estimated by the full KF model were <1 km away from the tracks derived from the LS model and 77% were <5 km.

## Discussion

Since the recent introduction of the Kalman filtering (KF) algorithm for the processing of satellite tracking data by the Argos system, the service providers have made this the default processing method for new transmitters (PTTs), giving the user the option to choose the Least Squares (LS) algorithm in alternative. The data processing of old PTTs that were already being processed with the LS algorithm remains unchanged, unless KF processing is requested, and stored data from 2008 onwards can be reprocessed using either method (albeit with additional processing costs). Processing of data with the new KF algorithm is bound to become more common as old PTTs end their life, and data processed with this algorithm will soon become the standard for Argos-based tracking.

State-space modelling approaches provide the statistical rigor needed in analysing animal movement data, but SSMs are not simple and require considerable care in their use [5]. Understanding the implications of using data processed with the new KF algorithm is essential when interpreting modelling results. To our knowledge this is the first time that performance of SSMs applied to KF tracking data has been directly validated with known locations of free-ranging animals. This was achieved by fitting the same model to Argos satellite locations obtained on 7 harbour seals processed with LS and KF algorithms and by comparing locations derived from each model against the “true” interpolated positions of the seals obtained by Fastloc GPS technology. In addition, the results of fitting the Bayesian switching state-space model (SSSM) to KF data were compared to those of LS models, using tracking data from 6 fin whales. Although in the latter case we could not assess the accuracy of model-derived locations, it enabled evaluating how SSSMs fit to KF data performed in relation to SSSMs fit to data processed with the LS algorithm, which until recently was the standard processing algorithm used to deliver satellite locations.

Our study shows that Kalman filtering consistently provided more estimated locations per animal track than the LS algorithm, supporting previous claims by the Argos service [19]. The increment in estimated locations was substantial for both species (fin whales: 75%; harbour seals: 56%). Compared to our findings, Boyd and Brightsmith [33] reported only a modest 28% increase in locations computed with the KF algorithm. However, their estimate is based on data obtained from static platforms, while our estimates and those from Argos come from free-ranging tagged animals. Stationary land tests are closer to the “ideal” conditions for satellite communications and are unlikely to adequately represent most of the problems known to affect the transmission of signals from satellite tags and/or the reception of messages at Argos satellites, especially for marine taxa. Understandably, the potential benefit of the KF method should be higher under circumstances (e.g. areas with limited satellite coverage) and for species more prone to transmission difficulties, and for which the frequency of uplinks is usually low. Not surprisingly, the major increase in estimated locations was for fin whales that typically have shorter surface intervals than harbour seals, and can be more adversely affected by wave wash due to improper antenna orientation and poor environmental conditions.

Like Boyd and Brightsmith [33], we also found that the majority of additional locations in KF data came from fixes with only 1message (Argos LC B) (fin whales: 29%; harbour seals: 33%) with a very slight increase in the proportion of locations with 4 or more messages (LC 2 and 3) observed only for fin whales. If, as a result of KF processing, tracks acquire a disproportionate number of locations with low spatial precision, this may impact the analysis and interpretation of animal movement data, particularly when this analysis is based on the raw satellite positions and doesn’t take into account variability in measurement errors. Implications could be even more severe if the gain in 1-message LC B locations is not homogeneously distributed along the track and depends, for instance, on the geographic location or behaviour of the animals, therefore being more prevalent in certain areas or during specific activities occurring in preferred habitats.

Our results demonstrate that the Jonsen et al. [8] SSM provided a good fit to the data processed with the KF dataset, despite the potentially increased autocorrelation in the location errors imposed by the KF algorithm. The greater spatial accuracy and precision of locations estimated from the KF model compared to those from the LS model was likely due to a combination of increased accuracy in KF-estimated locations and the higher temporal resolution of the KF data.

Although the overall difference in mean errors between the two algorithms appeared small (mean error in LS models was 3.5±3.0 compared to 2.9±2.9 in KF model) the model fit to KF data improved the accuracy of seal trips by 27% over the LS model. The linear mixed-effects model indicated that, despite significant variations in trip accuracy, errors in locations predicted for LS trips were significantly larger than those predicted for KF trips. For both models the largest deviances from true locations occurred along the east/west axis. This is not unexpected since Argos location errors are strongly biased towards the longitudinal component, regardless of the processing algorithm [1], [3], [31]–[33], and the SSM does not explicitly account for this directional bias. However, we found no evidence of the non-uniform distribution of extreme errors documented in other studies [3] suggesting that the model was able to handle this problem.

Tracks reconstructed from the models applied to KF and LS data provided faithful representations of the true seal trajectories measured with Fastloc GPS. However, the LS track tended to deviate more from the true track when seals were making short displacements and frequently changing direction. This is likely due to the correlated random walk model employed in the KF algorithm which would tend to smooth out uncommonly large changes in direction and/or displacement. As a result, LS locations tended to spread over a wider area compared to the KF. This was a common feature to several LS modelled tracks that can have major implications if these data are used to calculate sizes of home ranges or ARS patches.

The SSMs were fit as hierarchical models to the LS and KF data, meaning that data from all seal trips were combined to estimate model parameters, leading to improved location estimates. We anticipate that larger errors would be obtained if models were fitted separately to each trip. Yet, there is no reason to expect that the hierarchical formulation behaved differently when applied to LS and KF data, so we consider that the comparison between algorithms remains valid.

We fitted the same observation equation to data processed with LS and KF methods, thus assuming that the new algorithm did not change substantially the distribution or magnitude of the errors. A recent study demonstrated that both LS and KF location errors are better described by a long-tailed lognormal distribution [33]. In the present work, errors were modelled with generalized t-distributions which are known to be robust to extreme values [8]. Boyd and Brightsmith [33] also compared mean errors in KF and LS processed locations showing these did not differ significantly for most location classes, except for LC 2, for which LS errors were about half the KF errors, and LC B, for which LS errors were nearly 4 times greater than KF errors. In contrast, Argos reported better accuracies with the KF method for locations computed with ≥4 (LC 2 and 3) and 2–3 messages (LC A and B) [19]. In any case, given the predominance of LC classes A and B in both our datasets, we suspect that fitting the same observation equation to LS and KF data might have resulted in an overestimation of KF errors relative to LS errors, and not the other way around.

Regardless of which processing method is used, our study showed that accuracy of modelled tracks was sensitive to precision of the raw input data. As the proportion of locations with poor precision increased, the ability of the SSMs to recover accurate locations was significantly worse. This is consistent with findings from other researchers that showed that high measurement error not only impacts accuracy and precision of locations estimated from state-space methods [20], [34] but can also affect our ability to discern behavioural patterns and quantify habitat use patterns [4], [26], [32], [35], [36].

On the other hand, we found no evidence that observation frequency and temporal resolution of Argos data influenced the magnitude of SSM errors, in contrast to a recent study that suggested that frequency and regularity of raw data may be as important as spatial precision for obtaining accurate estimates of locations from state-space methods [20]. There are two main reasons for the different results between our analysis and that of Breed et al. [20]. First, Breed’s analysis of model accuracy was based on a reduced number of simulated tracks to which were imposed different observation frequencies and temporal gaps spanning a much larger range than the number of Argos locations and time steps observed in our seal data (see Table S1). Second, in Breed’s study a separate SSM was fit to each simulated track while we adopted a hierarchical approach. By combining information from all trips to estimate model parameters, potential effects of between-trip data quality likely were lessened and more accurate location estimates were obtained for all trips.

Our results strongly suggest that application of SSSM to the whale tracking data processed with the KF algorithm was appropriate and that models fitted well. Estimated parameters from KF models were very similar across all tracks and to parameters from the LS model despite the fact that models were fitted separately to each whale LS/KF-processed dataset.

Paths inferred from both models were also similar, with most of the locations from the reduced KF model falling within the 95% probability ellipses of locations estimated from the LS model, and the majority of locations from the full KF model being close to the whale tracks inferred by the LS model. Similar to what was observed for the seal data, the longitudinal bias in Argos errors caused the reduced KF locations to differ more from their paired LS positions in the east/west than in the north/south axis.

The estimated precision of locations inferred from the SSSM fit to the reduced KF data was higher for 5 out of 6 whale tracks, as indicated by the lower average width of the credible limits. However, the KF model behaved significantly worse than the LS model in the case of the whale track (#80716) for which less than 2 satellite positions were received per day. This cannot be accounted for by variations in Argos location classes because 28 of 29 positions were assigned the same class in both datasets. A close inspection of the raw KF and LS data indicates that the poorer performance of the reduced KF model was likely associated with the highly tortuous whale path evident in the KF data (and not in the LS data) and caused by the way the data regularization approach used in the SSSM’s observation model dealt with this tortuosity. Because the interval between raw satellite positions was considerably longer than the 3-hourly interval at which the SSSM positions were being estimated, raw positions have more weight on model estimates as the model “forces” derived locations to exactly match raw satellite positions. Such an effect tends to be more pronounced with decreasing linearity of the tracks [37], explaining why uncertainty in the model estimates was greater for the more sinuous KF path and the higher discrepancy in relation to the LS path.

It should be stressed that the application of the KF algorithm increased the total number of locations in this whale track from 29 to 108 (see Table S2), resulting in a remarkable decrease in the uncertainty of SSSM location estimates (average 95% CL width: 86.0±69.5 km) when compared to the LS model. Differences in the remaining tracks were less pronounced but the KF processing algorithm produced an overall increase in number of locations obtained and a decrease in the uncertainty of SSSM estimates.

Estimates of behavioural mode from the KF model agreed well with inferences from the LS model – with 94% of whale locations being assigned the same behavioural category in both models – indicating that the KF algorithm did not introduce appreciable changes in the ability of the SSSM to recover latent behaviours from satellite positions.

These results lead us to conclude that application of widely-used Bayesian state-space models [5] to Argos satellite locations processed with a KF method is appropriate and, as was the case of the SSM fit to harbour seal data, can produce more reliable location estimates than when LS data are used to fit the same models. Also, behavioural modes could be equally well detected from SSSM fit to whale tracking data processed with KF and LS methods. Since the KF algorithm generally yields more positions and longer tracks, there may be clear advantages in using the KF model over the LS model. This is especially true in telemetry studies of species that spend prolonged periods underwater or under dense vegetation cover, for which the number of daily fixes is generally low, precluding examination of movement and behaviour of animals in more detail. However, as seen here, the KF algorithm can increase the number of positions of lower precision (LC B) by nearly 30%, which in turn can degrade accuracy of modelled tracks. Even with LC B positions estimated by the KF method being several times more accurate than LS locations of equal class [19],[33], when accuracy and precision are critical for the analysis, researchers may consider removing 1-message positions before fitting state-space models.

## Supporting Information

Table S1 **GPS, Least Squares (LS) and Kalman filtered (KF) data obtained for each seal trip.**
(DOCX)

Table S2 **Argos Least Squares (LS) and Kalman filtered (KF) data obtained for each fin whale used to fit the switching state-space models.**
(DOCX)

Table S3 **Parameter estimates from the best fitting linear mixed-effects model for errors in modelled locations.**
(DOCX)
