# Supplementary material for: Assessing Performance of Bayesian State-Space Models Fit to Argos Satellite Telemetry Locations Processed with Kalman Filtering
Source: PLoS One. 2014 Mar 20;9(3):e92277. doi: 10.1371/journal.pone.0092277 (PMC3961316; doi:10.1371/journal.pone.0092277)
Supplement: Table S1 — GPS, Least Squares (LS) and Kalman filtered (KF) data obtained for each seal trip. (DOCX) [file pone.0092277.s001.docx]

| Seal | Trip | GPS data | | | LS-Argos data | | | KF-Argos data | | |
| --- | --- | --- | --- | --- | --- | --- | --- | --- | --- | --- |
|  |  | Trip duration (h) | N^[[1]](#endnote-2)^ | Time step (h)^[[2]](#endnote-3)^ | N^*^ | Time step (h)^†^ | LC 0-B^[[3]](#endnote-4)^ | N^*^ | Time step (h)^†^ | LC 0-B^‡^ |
| 1545 | 11 | 54 | 28 | 2.0 | 42 | 1.3 | 76.2 | 66 | 0.8 | 80.3 |
|  | 21 | 70 | 25 | 2.9 | 46 | 1.5 | 65.2 | 66 | 1.1 | 74.2 |
|  | 23 | 69 | 21 | 3.5 | 34 | 2.0 | 41.2 | 43 | 1.6 | 48.8 |
|  | 27 | 87 | 50 | 1.8 | 54 | 1.6 | 51.9 | 66 | 1.3 | 53.0 |
|  | 28 | 49 | 32 | 1.6 | 37 | 1.3 | 43.2 | 45 | 1.1 | 55.6 |
| 28503 | 11 | 56 | 36 | 1.6 | 44 | 1.3 | 81.8 | 68 | 0.8 | 88.2 |
|  | 18 | 42 | 31 | 1.4 | 31 | 1.4 | 77.4 | 46 | 0.9 | 82.6 |
|  | 19 | 59 | 43 | 1.4 | 41 | 1.5 | 85.4 | 63 | 0.9 | 88.9 |
|  | 23 | 30 | 31 | 1.0 | 33 | 0.9 | 87.9 | 47 | 0.6 | 87.2 |
|  | 42 | 46 | 23 | 2.1 | 33 | 1.3 | 72.7 | 55 | 0.8 | 80.0 |
| 43844 | 4 | 96 | 33 | 3.0 | 30 | 3.3 | 66.7 | 54 | 1.8 | 79.6 |
|  | 8 | 47 | 30 | 1.6 | 32 | 1.5 | 50.0 | 43 | 1.1 | 55.8 |
|  | 14 | 39 | 35 | 1.1 | 31 | 1.3 | 61.3 | 45 | 0.9 | 71.1 |
|  | 16 | 70 | 63 | 1.1 | 61 | 1.2 | 49.2 | 77 | 0.9 | 57.1 |
|  | 22 | 81 | 44 | 1.9 | 44 | 1.9 | 65.9 | 55 | 1.5 | 72.7 |
| 43871 | 7 | 97 | 77 | 1.3 | 77 | 1.3 | 87.0 | 124 | 0.8 | 91.9 |
|  | 8 | 63 | 57 | 1.1 | 43 | 1.5 | 86.0 | 76 | 0.8 | 90.8 |
|  | 13 | 82 | 76 | 1.1 | 58 | 1.4 | 82.8 | 102 | 0.8 | 85.3 |
|  | 19 | 67 | 63 | 1.1 | 48 | 1.4 | 83.3 | 79 | 0.9 | 88.6 |
| 120346 | 24 | 50 | 41 | 1.2 | 47 | 1.1 | 74.5 | 65 | 0.8 | 80.0 |
|  | 25 | 38 | 43 | 0.9 | 48 | 0.8 | 81.3 | 68 | 0.6 | 86.8 |
|  | 26 | 29 | 34 | 0.9 | 37 | 0.8 | 75.7 | 51 | 0.5 | 82.4 |
|  | 30 | 45 | 37 | 1.2 | 44 | 1.0 | 75.0 | 61 | 0.7 | 82.0 |
|  | 32 | 26 | 21 | 1.3 | 33 | 0.8 | 60.6 | 37 | 0.7 | 62.2 |
| 120349 | 3 | 91 | 18 | 5.4 | 39 | 2.4 | 87.2 | 62 | 1.5 | 95.2 |
|  | 4 | 100 | 20 | 5.2 | 35 | 2.9 | 88.6 | 69 | 1.5 | 95.7 |
|  | 5 | 83 | 20 | 4.4 | 41 | 1.9 | 90.2 | 97 | 0.8 | 95.9 |
|  | 6 | 49 | 20 | 2.6 | 34 | 1.5 | 88.2 | 79 | 0.6 | 94.9 |
| 120350 | 3 | 108 | 35 | 3.2 | 51 | 2.2 | 72.5 | 83 | 1.3 | 83.1 |
|  | 4 | 73 | 29 | 2.6 | 39 | 1.9 | 79.5 | 78 | 1.0 | 87.2 |
|  | 5 | 66 | 58 | 1.2 | 72 | 0.9 | 81.9 | 113 | 0.6 | 90.3 |
|  |  |  |  |  |  |  |  |  |  |  |
| Total |  | 1957 | 1174 | 1.7 | 1339 | 1.5 | 73.8 | 2083 | 0.9 | 81.8 |

1. N: Number of locations. [↑](#endnote-ref-2)
2. Time step: Average time between locations per trip. [↑](#endnote-ref-3)
3. Proportion of locations of LC 0, A and B. [↑](#endnote-ref-4)
