# Supplementary material for: Assessing Performance of Bayesian State-Space Models Fit to Argos Satellite Telemetry Locations Processed with Kalman Filtering
Source: PLoS One. 2014 Mar 20;9(3):e92277. doi: 10.1371/journal.pone.0092277 (PMC3961316; doi:10.1371/journal.pone.0092277)
Supplement: Table S2 — Argos Least Squares (LS) and Kalman filtered (KF) data obtained for each fin whale used to fit the switching state-space models. (DOCX) [file pone.0092277.s002.docx]

|  | LS-Argosdata | | | KF-Argos data | | |
| --- | --- | --- | --- | --- | --- | --- |
| Whale | Track duration (days) | N^[[1]](#endnote-2)^ | Mean nº positions/day | Track duration (days) | N^*^ | Mean nº positions/day |
| 80702 | 18 | 213 | 11.8 | 18 | 253 | 14.1 |
| 80704 | 23 | 258 | 11.2 | 34 | 333 | 12.3 |
| 80707 | 19 | 215 | 11.3 | 19 | 254 | 13.4 |
| 80713 | 3 | 17 | 5.7 | 3 | 30 | 10.0 |
| 80716 | 18 | 29 | 1.6 | 18 | 108 | 6.8 |
| 89969 | 52 | 1604 | 30.9 | 55 | 2125 | 39.4 |
|  |  |  |  |  |  |  |
| Total | 133 | 2336 | 12.1 | 147 | 3103 | 16 |

1. N: Number of locations. [↑](#endnote-ref-2)
