# Supplementary material for: Assessing Performance of Bayesian State-Space Models Fit to Argos Satellite Telemetry Locations Processed with Kalman Filtering
Source: PLoS One. 2014 Mar 20;9(3):e92277. doi: 10.1371/journal.pone.0092277 (PMC3961316; doi:10.1371/journal.pone.0092277)
Supplement: Table S3 — Parameter estimates from the best fitting linear mixed-effects model for errors in modelled locations. (DOCX) [file pone.0092277.s003.docx]

|  |  | Fixed effects | | | Random effect | |
| --- | --- | --- | --- | --- | --- | --- |
| Variable | Factor | Estimate | SE | *P* | Variance | SD |
| Trip |  |  |  |  | 0.09 | 0.303 |
| Intercept |  | -1.780 | 0.393 | <0.001 |  |  |
| Algorithm | LS | 0.439 | 0.078 | <0.001 |  |  |
| Proportion LC 0-B |  | 2.865 | 0.483 | <0.001 |  |  |
